# Avoid reinventing the wheel: implementation of the Ottawa Clinic Assessment Tool (OCAT) in Internal Medicine

**DOI:** 10.1186/s12909-018-1327-7

**Published:** 2018-09-20

**Authors:** Samantha Halman, Janelle Rekman, Timothy Wood, Andrew Baird, Wade Gofton, Nancy Dudek

**Affiliations:** 1 Department of Medicine, the University of Ottawa, The Ottawa Hospital General Campus, 501 Smyth Road, Box 209, Ottawa, Ontario K1H 8L6 Canada; 2 Department of Surgical Education, the University of Ottawa, The Ottawa Hospital Civic Campus, Loeb Research Building - Main Floor WM150b, 725 Parkdale Avenue, C/O Isabel Menard, Ottawa, Ontario K1Y 4E9 Canada; 3 Department of Innovation in Medical Education, Faculty of Medicine, the University of Ottawa, 850 Peter Morand Crescent (Room 102), Ottawa, Ontario K1G 5Z3 Canada; 4 Department of Medicine, the University of Ottawa, The Ottawa Hospital Parkdale Campus, Room 162, 1053 Carling Avenue, C/O Odile Kaufmann, Ottawa, Ontario K1Y 4E9 Canada; 5 Department of Surgical Education, the University of Ottawa, Ottawa Hospital - Civic Campus, Suite J15, 1053 Carling Avenue, Ottawa, Ontario K1Y 4E9 Canada; 6 Department of Medicine, the University of Ottawa, The Rehabillitation Centre. 505 Smyth Road, Ottawa, Ontario K1H 8M2 Canada

**Keywords:** Workplace-based assessment, Entrustment alignment, Ambulatory setting

## Abstract

**Background:**

Workplace based assessment (WBA) is crucial to competency-based education. The majority of healthcare is delivered in the ambulatory setting making the ability to run an entire clinic a crucial core competency for Internal Medicine (IM) trainees. Current WBA tools used in IM do not allow a thorough assessment of this skill. Further, most tools are not aligned with the way clinical assessors conceptualize performances. To address this, many tools aligned with entrustment decisions have recently been published. The Ottawa Clinic Assessment Tool (OCAT) is an entrustment-aligned tool that allows for such an assessment but was developed in the surgical setting and it is not known if it can perform well in an entirely different context. The aim of this study was to implement the OCAT in an IM program and collect psychometric data in this different setting. Using one tool across multiple contexts may reduce the need for tool development and ensure that tools used have proper psychometric data to support them.

**Methods:**

Psychometrics characteristics were determined. Descriptive statistics and effect sizes were calculated. Scores were compared between levels of training (juniors (PGY1), seniors (PGY2s and PGY3s) & fellows (PGY4s and PGY5s)) using a one-way ANOVA. Safety for independent practice was analyzed with a dichotomous score. Variance components were generated and used to estimate the reliability of the OCAT.

**Results:**

Three hundred ninety OCATs were completed over 52 weeks by 86 physicians assessing 44 residents. The range of ratings varied from 2 (I had to talk them through) to 5 (I did not need to be there) for most items. Mean scores differed significantly by training level (*p* < .001) with juniors having lower ratings (M = 3.80 (out of 5), SD = 0.49) than seniors (M = 4.22, SD = − 0.47) who had lower ratings than fellows (4.70, SD = 0.36). Trainees deemed safe to run the clinic independently had significantly higher mean scores than those deemed not safe (*p* < .001). The generalizability coefficient that corresponds to internal consistency is 0.92.

**Conclusions:**

This study’s psychometric data demonstrates that we can reliably use the OCAT in IM. We support assessing existing tools within different contexts rather than continuous developing discipline-specific instruments.

**Electronic supplementary material:**

The online version of this article (10.1186/s12909-018-1327-7) contains supplementary material, which is available to authorized users.

## Background

Residency training programs around the world are shifting from traditional time-based curricula to approaches organized around competencies and oriented towards outcome abilities [1]. The emergence of competency-based medical education (CBME) is a reflection of this restructuring of our educational paradigms [2].

Resident physicians have finished medical school and complete the remainder of their training in the workplace. In Canada, the Royal College of Physicians and Surgeons of Canada (RCPSC) objectives of training in the specialty of Internal Medicine (IM) define the internist as a physician who cares for hospitalized and ambulatory patients thus making the ambulatory clinic setting an important workplace environment for IM trainees [3]. Given that the majority of health care is now delivered within the ambulatory setting [4], we must ensure that our trainees are given the opportunity to become competent at providing care within this setting.

CBME curricula must incorporate effective workplace based assessment (WBA) programs [5]. To assess trainees in the ambulatory setting, programs are currently using a number of available WBA tools but many have limitations. The most studied tool for assessing a single patient encounter is the Mini-Clinical Evaluation Exercise (mini-CEX), with many studies demonstrating its reliability and validity properties in varied clinical contexts [6–8]. While the mini-CEX will continue to be an essential WBA tool, it does not assess some crucial clinic skills such as time management or prioritizing cases within a busy outpatient setting. Although single-patient encounter assessments remain important, it is often more feasible in WBA to include care provided to groups of patients rather than individuals [8]. Daily Encounter Cards (DECs) have been implemented in various settings and allow regular documented assessments which may span across more than one patient encounter [9] but reliability issues have been described [10]. To sample across a range of clinical encounters, many programs use the In-training Evaluation Report (ITER) as an aggregate WBA tool [10]. However, issues such as ITERs not being completed by anyone who has directly observed the resident or being filled several weeks after the resident completes the rotation [11] have contributed to the overall poor reliability and lack of validity evidence for ITERs [12].

It is necessary for us to be able to assess an IM trainee’s ability to manage a clinic but our current assessment methods are not well aligned with this goal. The Ottawa Clinic Assessment Tool (OCAT) is a competency-based WBA tool that was recently developed to assess daily performance in outpatient clinics for surgery residents [13]. The OCAT uses an entrustability rating scale which was developed for the Ottawa Surgical Competency Operating Room Evaluation (O-SCORE), a WBA tool which has been shown to have good reliability and validity evidence [14, 15]. Entrustability rating scales or entrustment anchored scales rely on the idea that physicians routinely ask themselves “Can I leave this resident alone?” [16–18]. Although many such tools were originally developed for procedural contexts [14, 15, 19, 20], recent work reveals that entrustment-based scales also apply to non-procedural specialties such as internal medicine [21, 22] and pediatrics [23]. Entrustment as it applies to WBA has been shown to improve rating discrimination and inter-rater reliability [24–27].

In reviewing the OCAT, it becomes apparent that none of the individual items are worded specifically for surgical trainees or patients. In other words, it would seem possible to use the OCAT in an IM clinic. Using existing tools across different contexts makes sense if it can be done with robust psychometric support as it would decrease the burden on educators to constantly develop specialty-specific tools. The aim of this study was to implement the OCAT and to collect psychometric data for its use in the IM ambulatory context.

## Methods

### OCAT description

The OCAT contains 11 items (9 mandatory and 2 optional, depending if technical skills were performed) rated on a 5-point scale and 2 yes/no items with regards to concerns with attitude/professionalism and ability to safely manage the clinic independently at a generalist level. These yes/no items are meant to be independent judgments from the rating on the items. Each of the 9 mandatory items is defined with a simple heading followed by key elements of this item. The 9 items are: history, physical exam, case presentation, differential diagnosis, management plan, patient/family communication, documentation within clinic, collaboration and time management of an entire clinic. As an example, the key elements of the last item are ‘able to economize time, manage interruptions, and modify time spent with individual patients appropriately.’ There are also 2 short-answer questions asking the rater to suggest one area for improvement and one area where the resident did well. Anchors on the scale ranged from 1 (*I had to do*) to 5 (*I did not need to be there*). Descriptive examples are provided for each anchor [13, 14]. The only modification we made to the OCAT before implementation in IM was to change the example specialties in the instructions (i.e. *urology, general surgery* were changed to *rheumatology*, *oncology*, *GIM*). All other items are worded exactly as they were in the original study [14]. The OCAT used in this study can be found in Additional file 1.

### Program description

Exposure to ambulatory care in our center occurs through three main clinical experiences: (1) a dedicated 4 week rotation (ambulatory block) where residents only attend clinics, (2) through the various subspecialty rotations (e.g. oncology) which typically will include some outpatient clinics on top of the mandatory inpatient work, and (3) through a longitudinal General Internal Medicine (GIM) clinic. Each clinic typically runs over one half-day and includes a mix of new consultations and follow-up visits.

The ambulatory block is composed predominantly of GIM and preoperative care clinics with a mix of subspecialty clinics based on trainee preference. The ambulatory block is reserved for second or third year trainees. Trainees may rotate through the subspecialty rotations at any point in their training although will typically have a first exposure either as first or second year residents with subsequent exposure primarily based on interest. The longitudinal clinic is reserved for trainees in their fourth and fifth year of training (dedicated to those in the GIM subspecialty program). The same case mix is seen in the longitudinal clinic with the exception that PGY4/5 s attend the same clinic on a weekly basis throughout the year which allows them to monitor patients longitudinally and build longer last patient-physician relationship. PGY5 residents will occasionally supervise PGY2/3 s rotating through their clinic (but their assessments are not captured in this study).

Within the IM program, residents are considered to be juniors as PGY1s and seniors as PGY2s and PGY3s. Residents who subsequently choose GIM as a subspecialty are considered fellows as PGY4s and PGY5s. In light of the current clinic allocation with regards to timing within training, the objectives of training for the ambulatory block are the same for PGY2s and PGY3s, and longitudinal clinic objectives are the same for PGY4s and PGY5s. Given this structure, we decided to group data for PGY1s (juniors), PGY2 and PGY3s (seniors), and PGY4 and PGY5s (fellows) for this study.

### Participants

In preparation for the implementation of a competency-based curriculum, the IM Residency Training Program at the University of Ottawa decided in the spring of 2015 to implement the OCAT as a replacement to a daily encounter card (DEC) for the ambulatory care block and the longitudinal clinic. As such, all residents enrolled in the IM and GIM programs used the OCAT and no active participant recruitment was required. We did not continue to collect DECs as there was significant concern for evaluation fatigue should both be required. Further, there was voiced dissatisfaction with the DEC from both faculty and residents.

In our current model, subspecialty rotations have various assessment methods. Individual rotations continued to use their choice of mandatory assessments but supervisors were asked to voluntarily also complete OCATs during these rotations. Raters received no specific training beyond the instructions written directly on the OCAT. Raters could choose how they wished to observe residents in clinic. Assessments were based on a combination of direct observation, indirect observation, case discussions and consultation note review.

### Data collection

All residents in the core IM program (*n* = 83) and GIM programs (*n* = 7) were considered for this study (*n* = 90). Data was collected over 12 months. Residents on the ambulatory block typically attend 4 clinics per week with the remainder of the time dedicated to academic activities. For this study, we required residents rotating through the ambulatory block to complete one OCAT per clinic. While on subspecialty rotations, residents attend a variable number of clinics per week. As such, we used reliability estimates from the original OCAT study to suggest a minimum of 3 forms per week during these rotations. GIM residents attend one longitudinal clinic per week. They were asked to submit one OCAT every month given that one resident is typically supervised by a smaller pool of preceptors thus potentially contributing to redundancy or assessment fatigue if one OCAT per clinic was required.

During the initial 8 months of the study period, paper copies of the OCAT were distributed to residents on the ambulatory block and in GIM longitudinal clinic. Residents rotating through subspecialty rotations were emailed instructions to print the OCAT and distribute to preceptors. All forms were returned by residents to the IM/GIM coordinators. This method of resident-driven distribution and collection of paper forms was the same as with our prior DEC. After 8 months, to facilitate data collection, the OCAT was made available electronically via the One45 platform. Data was subsequently collected via One45 with the exception of subspecialty rotations that remained paper-based. Data was anonymized and all resident personal identifying data was removed except for level of training.

### Analysis

The psychometric characteristics of the scale were determined. Descriptive statistics including item means, standard deviation and range were calculated. An item analysis including calculation of item-total and inter-item correlations was also carried out. An analysis of variance was conducted on the ratings using G-String and UrGenova to generate variance components (generalizability analysis (*g*-study)) which were subsequently used to produce estimates of the reliability of the ratings. For the generalizability analysis, OCAT forms were nested in resident and resident nested in training level. Training level was crossed with items. Although rater is a potential variable in this study, it proved to be difficult to include in the g-study. Some raters provided ratings for a given resident on more than one clinic, other raters never saw particular residents. In addition, there was only one rater per clinic, therefore the two variables are confounded and difficult to pull apart. For these reasons, we did not explicitly include rater in the design. The form variable that was included captures the influence of rater but caution is needed in interpreting due to the confounding. To examine a training level effect in more detail, a subsequent analysis of mean OCAT scores (averaged over 9 items) was conducted using a between subject ANOVA with training level (junior, senior, fellows) as a between subject factor. T-tests were used analyze differences. Mean scores between PGY2 and PGY3 residents were compared in a post hoc analysis to assess whether raters being unblinded to training level contributed to scores. Mean OCAT scores by training level and the safety for independent practice item were analyzed using a between subject factorial ANOVA. A chi-square test of independence was also conducted between training level and the safety for independence practice item.

## Results

A total 452 OCATs were completed over 52 weeks by 86 physicians assessing 44 residents. After removing forms with missing data, a total of 390 forms for 44 residents remained for an average of 8.86 forms per resident (range 1 to 30). A total of 62 forms were collected by juniors (PGY1s), 288 by seniors (PGY2 and PGY3s) and 40 by fellows (PGY4 and PGY5s). Slightly more forms were collected from the ambulatory block or GIM longitudinal clinics (*n* = 215) as opposed to subspecialty rotations (*n* = 175). The return rate of forms on the ambulatory block was 65% and did not differ between the paper-based format versus the electronic format (66% vs 63%, *p* = 0.62).

### Descriptive statistics

Table 1 shows the descriptive statistics for each of the 9 mandatory items rated on the 5-point scale. Less than 5% of completed OCATs had data on the two optional technical skills items so these items were excluded from further analysis. No residents were flagged for professionalism concerns. The mean rating for each item ranged from 3.93 to 4.42. The item-total correlations (ITCs) were high, ranging from 0.69 to 0.83, indicating that ratings on the items were similar. Inter-item correlations ranged from *r* = 0.45 to *r* = 0.78 (see Table 5 in Appendix 2).

**Table 1:** Descriptive statistics for the OCAT in Internal Medicine

OCAT Item	Rating		Range		ITCs
	Mean	SD	Min	Max	
History	4.26	0.63	2	5	0.79
Physical Exam	4.24	0.62	2	5	0.75
Case presentation	4.30	0.64	2	5	0.83
Differential Dx	4.06	0.67	2	5	0.76
Management plan	3.93	0.70	2	5	0.71
Communication	4.41	0.60	3	5	0.76
Documentation	4.35	0.59	3	5	0.73
Collaboration	4.42	0.60	1	5	0.69
Time management	4.31	0.65	3	5	0.74

*SD* Standard deviation, *Min* Minimum rating, *Max* Maximum rating, *ITCs* Item-total correlations

### Generalizability analysis

The results of the generalizability analysis are provided in Table 2. The facets included in the analysis in forms (f), residents (r), training level (t) and items (i). Training level accounts for 24% of the variance which indicates that there are differences between the ratings for the three training levels. The facet *r*:*t* accounts for 6% of the variance in ratings and indicates that within a given training level, residents had similar ratings. Within a resident, however, there were significant variations in OCAT ratings because the *f*:*r*:*t* facet accounted for 32% of the variance in ratings. Facets involving items (*i*, *ti* and *ri*:*t*) did not account for a large amount of variability indicating that item ratings were similar.

**Table 2:** Generalizability analysis

Effect	σ ^2^	%
*t*	0.12	24
*i*	0.02	4
*r:t*	0.03	6
*f:r:t*	0.13	32
*ti*	0.01	1
*ri:t*	0.01	2
*fi:r:t*	0.15	31

*f* Forms, *r* Resident, *t* Training level, *i* Item, *σ 2* variance component, *%* % variance

Using the variance components reported in Table 2, the *g*-coefficient for the scale was 0.61. It is also possible to derive a *g*-coefficient that corresponds to internal consistency. The resulting coefficient was 0.92. The formula is shown in Appendix 1.

### Effect of training level

There was a significant main effect of training level (*F*(2,387) = 50.48, *p* < .001, partial eta square = 0.21) with post-hoc t-test (least square difference) showing that scores for juniors are lower than all others (*p* < .001) and seniors are lower than fellows (*p* < .001) as demonstrated in Table 3. A between subject ANOVA was repeated for each individual OCAT item with the same pattern of scores of juniors < seniors < fellows (all *p* < 0.05) emerging (Table 6 in Appendix 3). Also, mean scores were not statistically different between PGY2 and PGY3s (PGY2 mean (*M*) = 4.19, standard deviation (*SD*) = 0.45; PGY3 *M* = 4.29, *SD* = 0.35; *p* = 0.24).

**Table 3:** Effect of training level on mean OCAT scores

Training level	Mean	SD	*N*
Juniors	3.80	0.49	40
Seniors	4.22	0.47	288
Fellows	4.70	0.36	62

Abbreviations*: SD* Standard deviation, *N* Number of forms

### Safety for independent practice

A printing error led to the 5-point scale being applied to the safety for independent practice question rather than a yes/no answer on 33 forms. This left 357 forms across 44 residents. Table 4 displays the mean OCAT scores as a function of training level and whether the raters judged the resident as being safe to run a clinic independently. There was a significant difference between mean scores of those residents who were rated “no” (4.07, *SD* = 0.46; *n* = 220) and those that received an answer of “yes” (4.53, *SD* = 0.47; *n* = 137) (*F*(1,351) = 42.98, *p* < .001, partial eta square = .11). There was a significant different between resident level (F(2,351) = 30.77, *p* < .011, partial eta square = 0.15).

**Table 4:** Is the learner safe to run this clinic independently?

	Mean	No			Mean	Yes		
		SD	*N*	%		SD	*N*	%
Juniors	3.59	0.49	21	54	4.07	0.35	18	46
Seniors	4.11	0.43	188	71	4.46	0.48	75	29
Fellows	4.33	0.32	11	20	4.84	0.25	44	80
Total	4.07	0.46	220	62	4.53	0.47	137	38

Abbreviations: *SD* Standard deviation*, N* Number of forms*, %* Percentage of group

Of note, a small number of junior trainees were rated as safe to run the clinic independently. Of the 18 forms where a junior resident was felt to be safe for independent practice, all came from subspecialty rotations (83% from one discipline) and more than 50% were from one particular rater. The above analysis was repeated with the outlier rater removed (Table 7 in Appendix 4) to demonstrate its effect clearly. There was again significant difference between mean scores of those residents who were rated “no” (4.07, *SD* = 0.46; *n* = 218) and those that received an answer of “yes” (4.57, *SD* = 0.47; *n* = 124) (*F*(1,336) = 29.25, *p* < .001, partial eta square = 0.09).

A chi-square test of independence was conducted between training level and safety for practice on all 357 forms. There was a statistically significant association between training level and safe to practice, χ2(2) = 52.10, p < .001, Cramer’s V = 0.38) with more fellows deemed safe as compared to seniors (Table 8 in Appendix 5).

## Discussion

The majority of health care in IM is delivered in the ambulatory setting but there are few tools designed specifically to assess a resident’s ability to competently manage a clinic. This study implemented the OCAT, a WBA tool with good validity evidence in the surgical setting [13], in IM clinics to determine whether it can function in a different clinical context. Our data demonstrates that the OCAT could very easily be adapted to the IM context without item alteration. This is an important strength of this study and is different from other studies where different scales were developed for different contexts [23]. The use of one scale across various contexts, after demonstrating it performs well from a psychometric perspective, will prevent raters from being burdened by the continuous introduction of new tools; this also ensures we are measuring what we intend to measure.

We were able to demonstrate that the OCAT can differentiate between training levels with juniors having lower scores than seniors and seniors lower scores than fellows. We were also able to demonstrate that lower scores were assigned to those residents that were not felt to be safe to run the clinic independently. Those nearing the end of training (fellows) were more likely to be deemed safe to practice independently. In reviewing the results, we noted that a small number of juniors were rated as safe to run the clinic which we felt was unlikely. When removing an outlier rater who contributed to more than 50% of these ratings, the proportion of juniors deemed safe to practice decreased from 46 to 27%. This is still a surprisingly high number and speaks to the need for targeted rater training. A study is underway to assess the impact of various forms of rater training for the OCAT.

The OCAT items appeared to be highly related with one another leading to high item-total correlations, correlations between items and internal consistency. This pattern of results is very similar to the data obtained in the original validation studies for the both the OCAT and O-SCORE [13, 14]. When interpreting these values, it is important to keep the formative goal of the OCAT in mind. Although items were highly correlated we favor keeping the OCAT intact as tool items have been shown to be a rich source for generating feedback and discussions [28]. Faculty development efforts will need to ensure that raters are however not clustering their ratings but rather paying attention to individual items on the form.

Another finding was that very few clinics involved an assessment of technical skills. Moving forward we will need to re-evaluate whether to include the technical skill items on all forms. For example, it may make sense to continue using technical skills items in a rheumatology clinic where certain procedures (i.e. joint injections) routinely take place but not in other specialty clinics. No professionalism concerns were reported on the OCAT during our study. This is similar to data from our prior DEC where professionalism was assessed on a five point scale and where no resident scored below expectations in the 2 years prior to the OCAT implementation. Potentially no concerns actually arose or possibly clinical supervisors do not feel the WBA tools are the best place to highlight these concerns.

The mean OCAT and item ratings were generally high. This pattern could be indicative of systematic rater errors such as an end of scale aversion. However, our data shows that raters used the low end of the scale for each item (Table 1). This demonstrates that although raters frequently gave higher ratings, they were willing to use the lower end of the scale. Further research is underway to study the residents’ and attending physicians’ perceptions of entrustability scales and obtain qualitative data on whether they are meeting their intended purpose.

Finally, the generalizability coefficient for the OCAT with 8.86 forms per person was 0.61. Typically, acceptable reliability coefficients would be in the order of 0.7 to 0.8 for low stake assessments [29]. In the current study the magnitude of the generalizability coefficient is within an acceptable range given that the purpose is for formative assessment and feedback. That said, the lower reliability can be attributed primarily to the difference between the variation within a particular resident (i.e. the forms) versus the variation between residents within a level. A number of factors could be contributing to this finding. First, residents rotated through many different subspecialties where there is a learning curve and therefore variation across the forms a resident received would be expected. To account for this, we would need to study the use of the OCAT over a period of time where stability would be expected in their performance such as the first week on a rotation. However, we would need enough participants to ensure an accurate sample size. Second, raters were not blinded to the training level of the residents and therefore that may have contributed to the low variation between residents. We are however encouraged that raters were not solely relying on known level of training given that mean scores were not statistically different between PGY2 and PGY3 residents. Given the smaller number of forms, we were unable to determine if this was also true between PGY4 and PGY5s. Further, researchers who developed O-SCORE were able to demonstrate that the tool could differentiate surgical trainee level when the rater was blinded [15] or unblinded [14] to training level. Blinding could be achieved by having faculty from different institutions assess our residents but would be incredibly costly and unrepresentative of our actual reality.

The OCAT return rate was lower than anticipated. The return rate from the ambulatory block was 65% which is similar to prior experience with our DEC. Subspecialty exposure to outpatient clinics will typically span from 2 to 4 weeks. We obtained an average of 5.5 forms per rotation which is less than the projected 9 if we average to 3 weeks per rotation. This was likely due to a number of factors including the reliance on residents for collecting forms and the voluntary basis on which we asked subspecialty supervisors to complete the OCAT on top of their rotation-specific assessments. Of important note, although we considered all residents eligible for the study (*n* = 90), it is possible that some residents did not have the opportunity to collect any forms during the 12 month period which overlapped two academic years.

## Conclusions

We have demonstrated that a clinic based WBA tool developed for one context can be applied to another with good psychometric data to support its use. The OCAT provides the opportunity for clinical supervisors to assess resident performance in IM clinics. The OCAT is well anchored in CBME and it helps raters convey an expert judgment of performance within an authentic context. Future work will include collecting further validity evidence for OCAT scores in the IM setting as well as an exploration of the effects of time and various formats of rater training.

### Additional file

Additional file 1: The Ottawa Clinic Assessment Tool (OCAT). (DOCX 17 kb)

## Appendix 1

Formulas used to calculate g-coefficients using variance components

$$ G(overall)=\frac{\sigma^2\left(r:t\right)+{\sigma}^2\left( ri:t\right)/{n}_i}{\sigma^2\left(r:t\right)+{\sigma}^2\left( ri:t\right)/{n}_i+{\sigma}^2\left(f:r:t\right)/{n}_f+{\sigma}^2\left( fi:r:t\right)/{n}_f{n}_i} $$

$$ G\left( internal\ consistency\right)=\frac{\sigma^2\left(r:t\right)+{\sigma}^2\left(f:r:t\right)}{\sigma^2\left(r:t\right)+{\sigma}^2\left(f:r:t\right)+{\sigma}^2\left( ri:t\right)/{n}_i+{\sigma}^2\left( fi:r:t\right)/{n}_i} $$

*Abbreviations: f = forms, r = resident, t = training level, i = item, σ* ^*2*^ *= variance component*

## Appendix 2

**Table 5:** Inter-Item Correlation Matrix

	Q1	Q2	Q3	Q4	Q5	Q6	Q7	Q8	Q9
Q1	1.00	0.73	0.78	0.65	0.58	0.63	0.63	0.52	0.60
Q2	0.73	1.00	0.73	0.67	0.54	0.57	0.57	0.49	0.56
Q3	0.78	0.73	1.00	0.72	0.62	0.70	0.58	0.59	0.58
Q4	0.65	0.67	0.72	1.00	0.73	0.57	0.51	0.50	0.56
Q5	0.58	0.54	0.62	0.73	1.00	0.54	0.53	0.45	0.58
Q6	0.63	0.57	0.70	0.57	0.54	1.00	0.64	0.65	0.62
Q7	0.63	0.57	0.58	0.51	0.53	0.64	1.00	0.63	0.63
Q8	0.52	0.49	0.59	0.50	0.45	0.65	0.63	1.00	0.66
Q9	0.60	0.56	0.58	0.56	0.58	0.62	0.63	0.66	1.00

Q = item

## Appendix 3

**Table 6:** Effect of training level on mean item OCAT scores

	Juniors (*n* = 40)		Seniors (*n* = 288)		Fellows (*n* = 62)	
	Mean	SD	Mean	SD	Mean	SD
History	3.70	0.69	4.23	0.57	4.77	0.42
Physical Exam	3.65	0.62	4.21	0.55	4.77	0.46
Case presentation	3.68	0.62	4.28	0.60	4.81	0.40
Differential Dx	3.50	0.68	4.03	0.62	4.55	0.50
Management plan	3.43	0.75	3.89	0.64	4.47	0.62
Communication	4.00	0.56	4.38	0.58	4.77	0.46
Documentation	4.05	0.55	4.31	0.57	4.69	0.53
Collaboration	4.18	0.68	4.38	0.59	4.79	0.41
Time management	4.05	0.71	4.27	0.64	4.68	0.47

Abbreviations: *SD* Standard deviation, *N* Number of forms

## Appendix 4

**Table 7:** Is the learner safe to run this clinic independently? (outlier removed)

	Mean	No			Mean	Yes		
		SD	*N*	%		*SD*	*N*	%
Juniors	3.58	0.46	19	73	4.05	0.50	7	27
Seniors	4.11	0.43	188	72	4.46	0.49	73	28
Fellows	4.33	0.32	11	20	4.84	0.25	44	80
Total	4.07	0.46	218	64	4.57	0.47	124	36

Abbreviations: *SD* standard deviation, *N* number of forms,*%* percentage of group

## Appendix 5

**Table 8:** Crosstabulation of training level by safe to run a clinic

	Safe to run clinic independently?	
	No	Yes
Juniors	−0.6	0.8
Seniors	2.0	−2.6
Fellows	−3.9	5.0

NB: Standard residuals shown with values above 1.96 being statistically significant

## Abbreviations

CBME: Competency-based medical education
DEC: Daily encounter cards
GIM: General internal medicine
IM: Internal Medicine
ITER: In-Training Evaluation Report
OCAT: Ottawa Clinic Assessment Tool
PGY: Post-graduate year
WBA: Workplace based assessment
