# Supplementary material for: Avoid reinventing the wheel: implementation of the Ottawa Clinic Assessment Tool (OCAT) in Internal Medicine
Source: BMC Med Educ. 2018 Sep 20;18:218. doi: 10.1186/s12909-018-1327-7 (PMC6148769; doi:10.1186/s12909-018-1327-7)
Supplement: Supplementary file 1 — The Ottawa Clinic Assessment Tool (OCAT). (DOCX 17 kb) [file 12909_2018_1327_MOESM1_ESM.docx]

Additional file 1: The Ottawa Clinic Assessment Tool (OCAT)

| Clinic: | Level: 1 2 3 4 5 | Staff: |
| --- | --- | --- |
| Resident Name: | | Date: |

**The purpose of this scale is to assess the trainee’s ability to safely and independently run a CLINIC IN YOUR SPECIALTY (i.e. rheumatology, oncology, GIM, etc.) at the level of a GENERALIST (i.e. certified graduate of a core IM residency program). With that in mind please use the scale below to rate each item, irrespective of the resident’s level of training. Base your rating on the trainee’s performance across the ENTIRE CLINIC (i.e. do not base your rating on only one specific patient encounter). Please complete the assessment *IMMEDIATELY* following completion of the clinic.**

1**—“I had to do”—**i.e., Requires complete guidance, unprepared to do, or had to do for them

2—“**I had to talk them through”—**i.e., Able to perform some tasks but requires repeated directions

3—“**I had to direct them from time to time**”—i.e., Demonstrates some independence, but requires intermittent prompting

4**—“I needed to be available just in case”—**i.e., Independence but needs assistance with nuances of certain patients and/or situations, unable to manage all patients, still requires supervision for safe practice

5**—“I did not need to be there”—**i.e., Complete independence, can safely manage a general clinic in your specialty

| **1. History**  Efficient data gathering | **1** | | **2** | **3** | **4** | **5** |  |
| --- | --- | --- | --- | --- | --- | --- | --- |
| **2. Physical Exam**  Efficient and accurate examination | **1** | | **2** | **3** | **4** | **5** |  |
| **3. Case Presentation**  Synthesis of history and physical, clear presentation | **1** | | **2** | **3** | **4** | **5** |  |
| **4. Differential Diagnosis**  Able to make a diagnosis and appropriately consider alternatives | **1** | | **2** | **3** | **4** | **5** |  |
| **5. Management Plan**  Able to develop relevant plan dependent on context and be decisive (i.e. appropriate investigations, procedures, etc) | **1** | | **2** | **3** | **4** | **5** |  |
| **6. Patient/Family Communication**  Effective, sensitive, and respectful communication skills (verbal & non-verbal), language appropriate to patient understanding, able to build rapport and trust | **1** | | **2** | **3** | **4** | **5** |  |
| **7. Documentation within Clinic**  Orders, prescriptions, forms, etc (may not include consultation report) | **1** | | **2** | **3** | **4** | **5** |  |
| **8. Collaboration**  Works well with and/or teaches other team members as appropriate (i.e. staff, student, other healthcare professional) | **1** | | **2** | **3** | **4** | **5** |  |
| **9. Time Management of Entire Clinic**  Able to economize time, manage interruptions, and modify time spent with individual patients appropriately | **1** | | **2** | **3** | **4** | **5** |  |
| **10. If Procedures Were Performed in Clinic: Not Applicable** ☐  **a. Technical Skills**  Safely and effectively performs appropriate clinical procedures | **1** | | **2** | **3** | **4** | **5** |  |
| **b. Situational Awareness**  Non-technical aspects of procedure (i.e. insight into patient experience, respects patient comfort) | **1** | | **2** | **3** | **4** | **5** |  |
| **11. Concerns with Attitude or Professionalism**  **(If yes please describe in suggestions for improvement below)** |  | | **No** |  | **Yes** |  |  |
| **12. Resident is safe to independently manage/run this clinic at a generalist level** |  | | **No** |  | **Yes** |  |  |
| **13. Give at least 1 specific aspect of clinic done well** |  | |  |  |  |  |  |
| **14. Give at least 1 specific suggestion for improvement** | | |  |  |  |  |  |
| **Staff Signature: _____________________________________** | |  |  |  |  |  |  |
